# Characterization of Arterial and Sinus Blood Flow Dynamics in the Rat Brain Using Phase-Contrast MRI

**DOI:** 10.1007/s10439-026-04056-2

**Published:** 2026-03-12

**Authors:** Sidy Fall, Kamel Abderrahim, Olivier Baledent

**Affiliations:** 1 University Centre for Health Research (CURS, PIRMPA), Jules Verne University of Picardy, Amiens, France; 2 Facing Faces Institute/CHIMERE UR 7516, Jules Verne University of Picardy, Amiens, France; 3 Centre Universitaire de Recherche en Santé, CURS-PIRMPA, site CHU Amiens, 1 Rond-Point du Professeur Christian Cabrol, 80000 Amiens, France

**Keywords:** Phase-contrast MRI, Flow, Pulsatility index, Artery, Venous-sinus, Rat

## Abstract

Phase-contrast magnetic resonance imaging (PC-MRI) is a widely used technique for measuring cerebral blood flow dynamics in humans. However, the application of this technique for velocity mapping of cerebral blood flow in rats has been very limited thus far. This study aimed to determine the accuracy of PC-MRI flow measurements and to establish reference flow values for the main intracerebral feeding and draining vessels in rats. PC-MRI velocimetry was conducted on a flow phantom and 12 adult Sprague Dawley rats. A semi-automatic segmentation was applied to the velocity images to enable the quantification of flows and the calculation of pulsatility index. PC-MRI flow measurements showed good agreement with the reference flow from the pump. The main findings of the in vivo measurements were as follows: the mean blood flow values in the internal carotid artery (ICA), external carotid artery (ECA), basilar artery (BA), and transverse sinuses were 9.6 ± 3.4, 5.7 ± 2.4, 2.0 ± 0.6, and 10.5 ± 3.6 ml/min, respectively. Mean ICA flow was higher than ECA flow, while the ICA pulsatility index was lower than the ECA pulsatility index. There was no significant difference in the mean cerebral inflow (combined ICA and BA flows) and cerebral outflow (left and right transverse sinus flows) measures. No differences in arterial or sinus flow were observed between the left and right sides of the brain. These reference flow values and pulsatility index may be useful for understanding flow dynamics in healthy and pathological conditions in rats.

## Introduction

Cerebral vascular pathologies are well-documented risk factors in the etiology of neurodegenerative diseases, hydrocephalus, and idiopathic intracranial hypertension [1–4]. Researchers have long used rat models of these diseases because they allow the underlying physiological mechanisms, course of the disease, and potential treatments to be studied during their relatively rapid life cycle [5]. Furthermore, the translation of research from rodent models to humans is facilitated by the overall similarities in the biological properties and vascular networks of their brains [6]. Transcranial Doppler sonography techniques are commonly used to examine vascular insufficiency in rat models of cerebral diseases by monitoring blood flow velocities in individual intracranial vessels [7, 8]. Although these techniques have been validated to provide real-time information about cerebral hemodynamics, they suffer from interobserver variability [9, 10] and it is difficult to quantify blood flow in deep vessels due to limitations imposed by brain tissue and skull density. Phase-contrast magnetic resonance imaging (PC-MRI), also known as velocity-encoded MRI, is a complementary neuroimaging technique that enables a non-invasive assessment of blood velocities throughout the cardiac cycle with relatively high spatiotemporal resolution. So far, flow quantification in venous-sinus and arterial systems in the rat brain has been very limited in published papers. Additionally, the sparse PCMRI flow data collected from rats has been analyzed to estimate the total cerebral blood flow [11] and to assess the blood flow velocity of intracranial microvessels [12] as well as extracranial macrovessels [13, 14]. However, these previous publications did not mention quantitative flow measurements or analysis of blood flow dynamics in major cerebral arteries and venous-sinus vessels. It is notable that the blood flow waveform can be characterized by its pulsatility, which is associated with vessel stiffness, microvascular structure and function, and pulse pressure level [15]. To the best of our knowledge, no studies have evaluated blood flow and characterized its dynamics in both the arterial and venous-sinus systems of rats using PC-MRI. The venous-sinus system is complex and, unlike the arterial network, exhibits greater anatomical variation. Furthermore, the venous-sinus system is crucial for maintaining normal flow and pressure in cerebral blood circulation. An increase in venous-sinus outflow resistance commonly causes drops in flow, which can affect cerebral blood microcirculation and perfusion in rats [16]. Therefore, it is essential to assess quantitative blood velocity measurements and flow properties in a normal rat population in order to understand and characterize pathological processes in preclinical rat models of vascular diseases [16–19].

The objectives of this study were threefold: (i) to evaluate the repeatability and accuracy of cine PC-MRI flow quantification at 7 Tesla using a flow phantom; (ii) to quantify blood flow velocities in the major intracerebral feeding and draining vessels in rats; and (iii) to determine blood flow pulsatility in these arterial and venous-sinus pathways.

## Materials and Methods

### Phantom Design

**Fig. 1:**
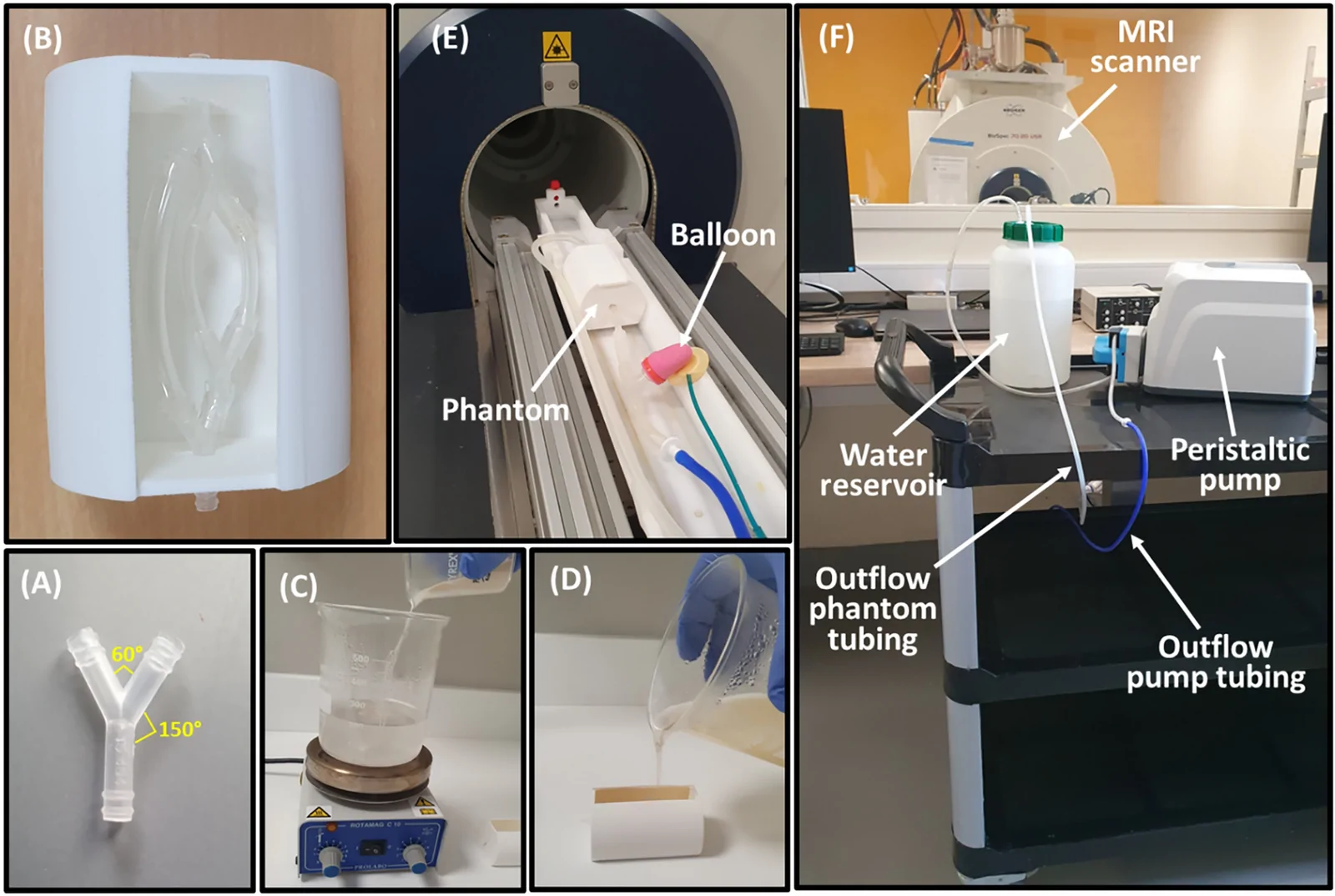
**A** A standard polypropylene Y-connector used to connect the different branches of the phantom, **B** shows the flow phantom inside its 3D-printed container, **C** and **D** are the process for preparing the agar gel: water was heated to ~ 80 °C, before the solution was mixed with the agar powder (2.5 g/100 mL) using a magnetic stirrer, **D** illustrates the next step in pouring the agar gel, **E** shows the phantom placed on the MRI bed. The balloon was positioned on a rodent breathing pillow in order to capture the pump cycles. The phantom’s inflow and outflow tubing were connected to the pump and reservoir, respectively, **F** shows the reservoir and the peristaltic pump, which were installed approximately 5 m from the center of the scanner and outside the MRI room

**Fig. 2:**
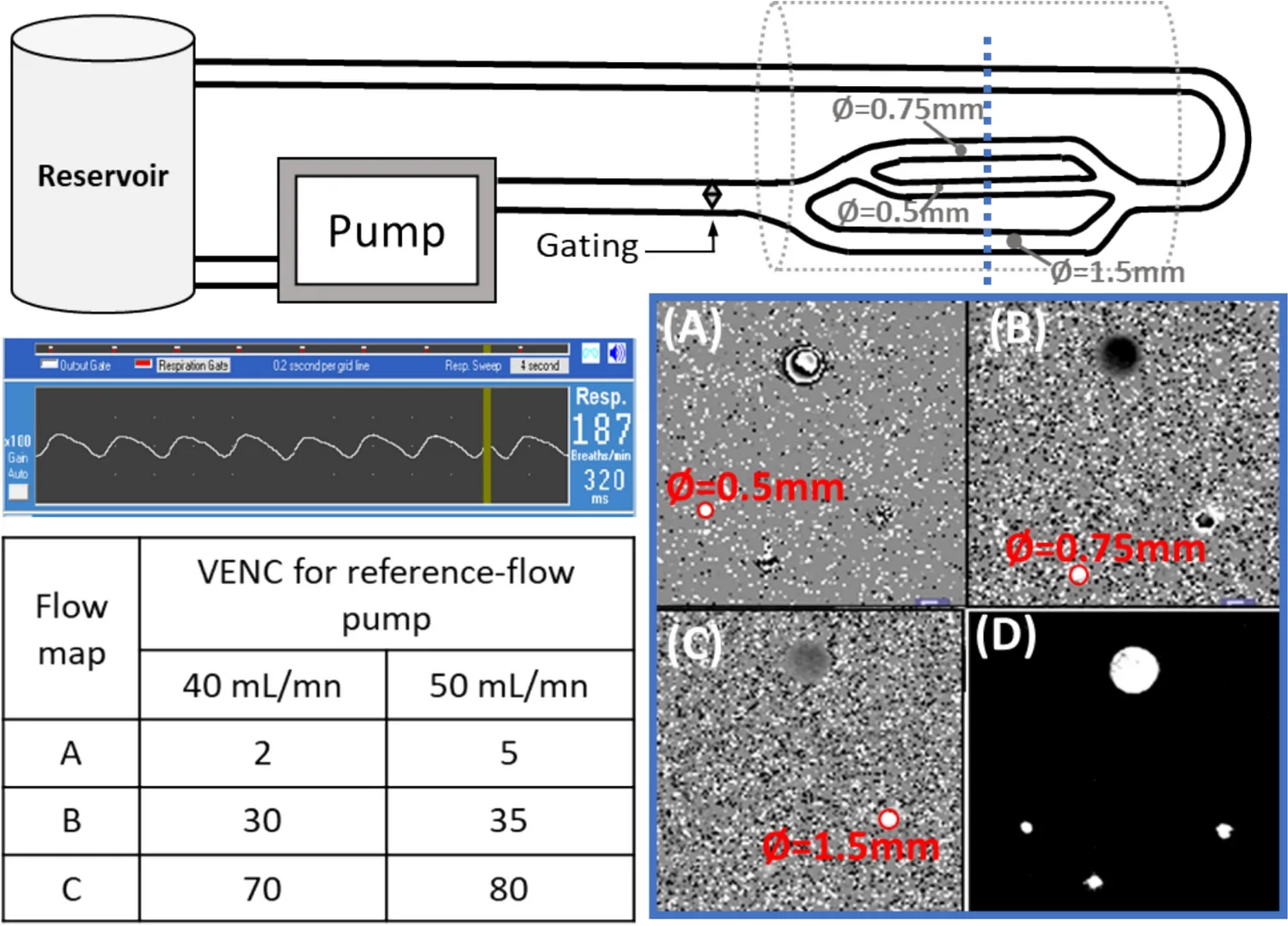
A schematic diagram of the water flow phantom is presented at the top. The table shows the velocity encoding (VENC) parameters adapted to the expected velocity for each of the two reference flows from the pump. Images, **A**–**C** show representative velocity maps of the upward flows (bright) within the three different branches of the silicon tubes. Red circles indicate regions of interest placed on the lumen area of the tubes. The blue dotted line shows the position of the PC imaging plane, **D** shows an example of a magnitude image displaying the tubes’ flow masks (bright). Above the table is an example of a gating signal captured by a monitoring module (SA Instruments, Inc., Stony Brook, NY, USA)

In the first part of this study, we used an in-house flow phantom to evaluate the accuracy and repeatability of our PC-MRI velocity measurements. The core of this phantom consisted of three silicone tubes with fixed internal diameters of 0.76, 1, and 1.50 mm, as specified by the manufacturer. The tubes were 45, 20, and 25 mm long, respectively. We used these tubes to evaluate low, medium, and high velocities, which were comparable to blood flow velocities in the vessels of interest in rats. A standard Y-connector (3.2 mm in inner diameter, Fig. 1A) was used to connect the different segments of the phantom, forming a simplified vascular geometry that resembles bifurcations of feeding arteries and draining sinuses. The tubes were glued to the Y-connectors and placed inside a cylindrical plastic container that measured 3.5 cm in diameter and 6.5 cm in length (Fig. 1B). The phantom was then cast in agar gel (Difco Agar^TM^, BD, USA) which gave a homogenous stationary background signal (Fig. 1C and D). A stable and calibrated pulsating flow pump, (MasterFlex L/S, Cole-Parmer Instrument Company, USA) was positioned outside the scanner room and supplied water to the flow phantom via a closed circuit containing a compliant balloon (Fig. 1E and F). A long tubing with an inner diameter of 4 mm was connected to the inlet and outlet of the phantom to supply and drain it through the water flow circuit. The periodic movements of the pump cycle were captured by a pressure sensor through the balloon and converted into a gating signal using a monitoring module (Small Animal Instruments, Inc. Stony Brook, NY, USA), see Fig. 2. The experiment was conducted at two flow rates: 40 and 50 mL/mn, corresponding to pulse rates of 150 and 187 beats per minute (BPM), respectively.

### Animal Protocol

This study was performed in accordance with European Union Directive 2010/63/EU on animal experiments and was also approved by our local animal ethics committee. Twenty female Sprague–Dawley rats (aged 6 ± 1.2 months, with a body weight 230–280 g) were anesthetized with isoflurane in air (5% for induction, then 1.5–2% to maintain a respiratory rate of 60–75 BPM). During imaging, the animals’ breathing rate was monitored using a breathing pillow (Small Animal Instruments Inc. Stony Brook, New York, USA) which was attached to their abdomens. Body temperature was monitored using a rectal probe and maintained within physiological conditions (36.5–37.5 °C) using a dedicated circulating water heating pad. Heart rates were monitored using electrodes applied to the paws. The animals were awake within 5 min following the acquisition, at which point they were returned to their home cage.

### MRI Protocol

#### MRI Hardware

Acquisitions were performed on a 7T Bruker scanner (BioSpec 70/20 USR, Bruker BioSpec, Germany) equipped with a maximum gradient of 360 mT/m and controlled with by Paravision 360 software. A volume coil of 86 mm inner diameter was used for radiofrequency transmission. For signal detection a 4-channel receive-only surface coil (Bruker, Germany) was used.

### Phantom: Imaging and Data Analysis

The phantom was positioned at the magnet isocenter for acquisition of the velocity images. These images were collected using a standard prospective-triggered PC-MRI sequence with the following parameters: repetition time/echo time (TR/TE) = 10/3.2 ms, flip angle (FA) = 30°, number of averages = 2, field of view (FOV) = 45 × 45 mm^2^, slice thickness = 1 mm, data matrix = 256 × 256. This yielded pixels with an in-plane resolution of 0.17 × 0.17 mm^2^. To avoid aliasing, the velocity encoding (VENC) was adjusted according to the expected flow range for each of the three tubes (see Table on Fig. 2). With the pump set to a flow rate of 40 ml/min, 38 phase images were acquired over 194 s. A total of 32 phase images were acquired during 163 s, with the pump set to a flow rate of 50 ml/min.

To evaluate consistent synchronization between the imaging sequence and the phases of the pump cycles, PC-MRI measurements were repeated five times with identical sequence parameters in a single session. The repeatability of the measurements (expressed as the percentage difference in measurements relative to their mean) was then evaluated using the coefficient of variation ($$\%CV=\frac{standard deviation}{mean}\times 100$$). To assess the accuracy of the measurements, the percentage difference between the PC-MRI flow measurement and the reference flow provided by the pump was calculated ($$percentage of accuracy=|\frac{reference flow - measured flow}{reference flow}|\times 100$$).

### Animal Imaging

**Fig. 3:**
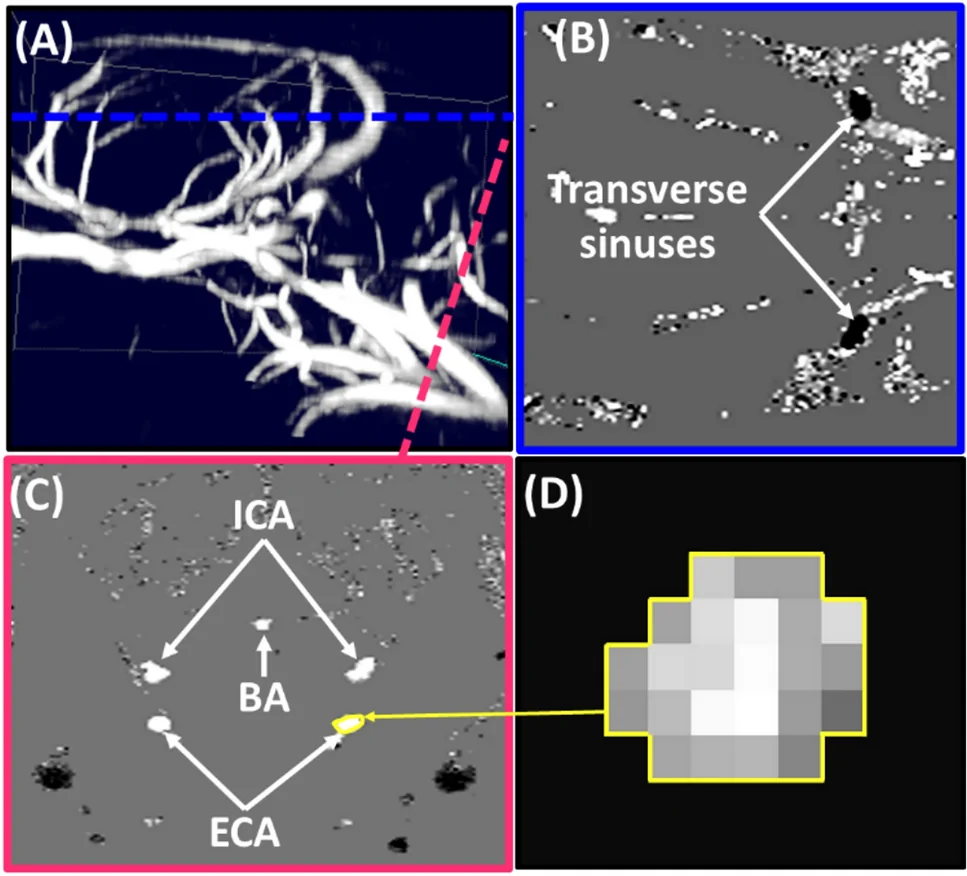
**A** A representative 3D maximum intensity projection (MIP) image obtained from PC angiograms of a rat. The dotted lines overlaid on the 3D MIP image indicate the locations of the slices for velocity imaging, **B** and **C** show examples of 2D velocity-encoded PC images, displaying dark signals from the transverse sinuses and bright signals from the internal carotid arteries (ICA), external carotid arteries (ECA), and basilar artery (BA), **D** shows an example of pixels corresponding to a segmented vessel

The animals were positioned on the prone position in the scanner bed. Prior to flow-velocity imaging, a three-dimensional (3D) phase-contrast angiography sequence was conducted using the following parameters: TR/TE = 16/4 ms, FA 25°, FOV = 32 × 32 × 20 mm^3^, data matrix = 256 × 256 × 64, and VENC = 20 cm/s. A total of 35 coronal images were obtained during 4.36 min. Maximum intensity projection (MIP) images obtained from angiograms were used to position the flow imaging planes at the most orthogonal orientation to the vessels of interest (Fig. 3A).

Flow images were acquired with a similar spatial resolution to that of the phantom experiment (0.17 × 0.17 × 1 mm^3^), with TR and TE were fixed at 10 and 2.8 ms, respectively. To minimize aliasing artifacts, the VENC parameter was adjusted from 60 to 80 cm/s for arterial flow, whereas it was fixed at 12 cm/s for the sinuses. For the arterial velocimetry, an oblique imaging plane was selected to intersect the internal (ICA) and external (ECA) carotid artery branches, approximately 2 mm above the inner common carotid artery bifurcation and passing through the basilar artery (BA). Velocity imaging of the transverse sinuses was conducted using an axial plane. All velocity images were acquired with a number of time frame adapted to the R-R interval duration. Scan times for the arterial and venous velocity mapping were approximately 2.5 and 1.3 min, respectively, depending on the animal’s heart and respiration rates.

### In Vivo Images Analysis

A specially developed software [20], programmed in Interactive Data Language (Research Systems, Inc., Boulder, CO) was used to segment vessel contours from the velocity images. To reduce the effect of partial volume, pixels with less than 50% of their area inside the segmented vessels were excluded from the analysis. Regions of interest were placed on the ICA, ECA, BA, and transverse sinuses using a semi-automatic segmentation approach (see Fig. 3B–D). Blood flows from these vessels were measured and displayed on graphs throughout the cardiac cycle. Blood flows from the ICA and BA were added together to produce a total intracranial arterial cerebral inflow curve. The flows from the left and right transverse sinuses were combined to measure the outflow of the brain sinuses. The arterio-sinus flow difference was calculated as the difference between these cerebral inflow and outflow. The maximum (${v}_{max}$), minimum (${v}_{min}$), and mean blood flow velocity (${v}_{mean}$) were measured from each vessels of interest during the cardiac cycle to calculate a pulsatility index (PI) using: $I=\frac{{v}_{max}-{v}_{mean}}{{v}_{mean}}$.

### Statistical Analysis

Statistical analysis was conducted using the IBM SPSS Statistics software (version 24.0, IBM Corp., Armonk, NY, USA). The measured flow values were expressed as means ± standard deviation (SD). Differences in flow measurements were assessed using the non-parametric paired *Wilcoxon* test. The threshold for significance was set at *p* < 0.05.

## Results

### In Vitro Phantom Imaging

Figures 2A–C show representative 2D velocity maps using VENC adapted for each of the three tubes. Phase aliasing effects caused by an underestimating the velocity peaks in the center pixels of the non-contoured tubes can be observed.

With a reference flow of 40 ml/min, the mean flows measured in the 0.76, 1, and 1.5 mm diameter tubes were 0.26 ml/min (%CV = 1.56), 6.32 ml/min (%CV = 0.34), and 32.11 ml/min (%CV = 0.56), respectively. The total flow calculated for these three tubes was 36.89 ml/min, with an accuracy of 8% relative to the reference flow from the pump.

For the second series of measurements, the reference flow from the pump was set to 50 ml/min. The mean flows measured in the three tubes were, respectively, 0.26 ml/min (%CV = 3.10), 6.32 ml/min (%CV = 0.61), and 39.76 ml/min (%CV = 2.95), respectively. The corresponding total flow was 47.19 ml/min, with an accuracy of 7% compared to the pump’s reference flow.

### In Vivo Flow Measurements

**Fig. 4:**
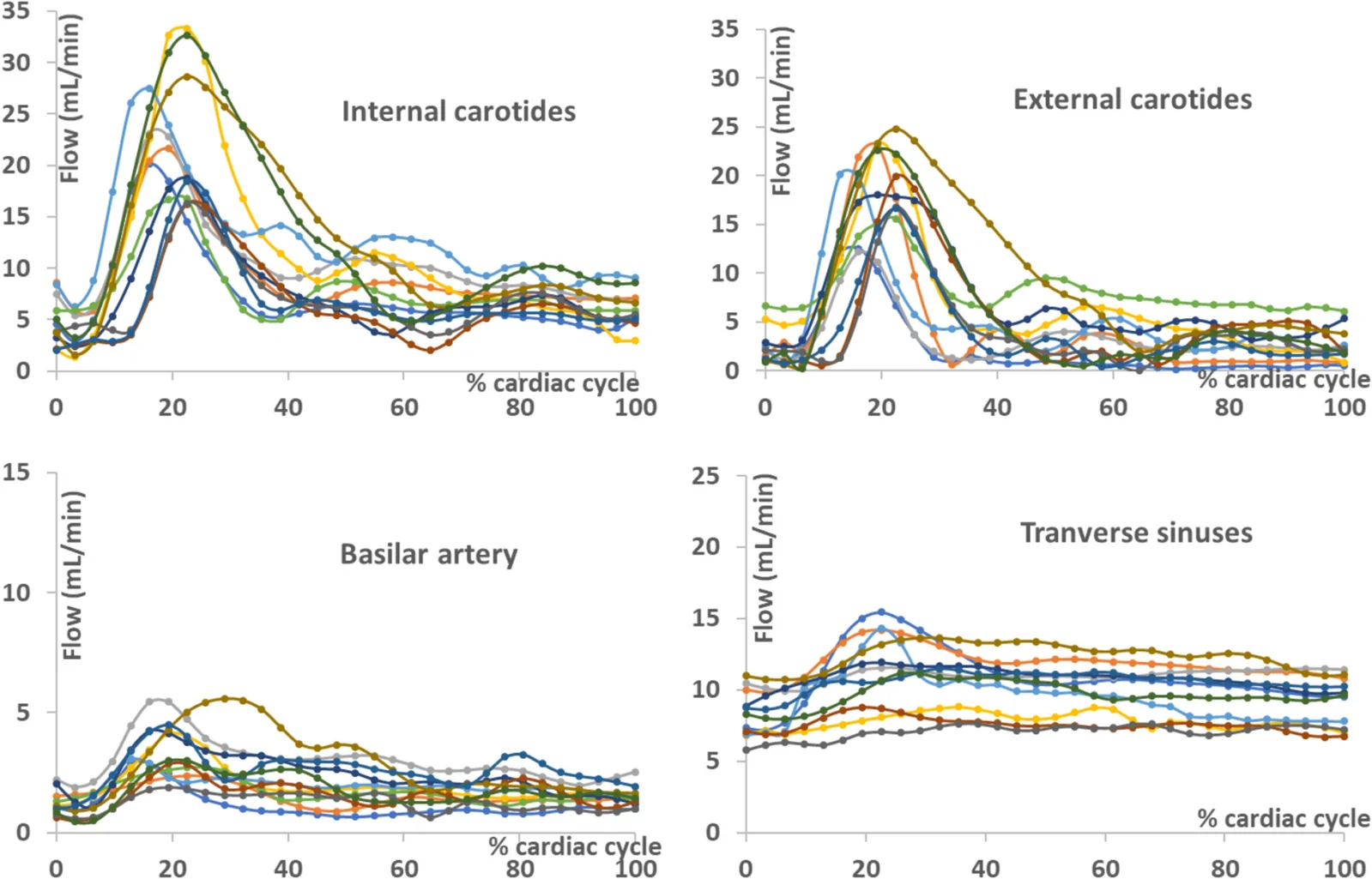
Flow curve profiles of the vessels of interest were extracted from our rat group. Each rat is represented by a colored curve. To account for differences in cardiac BPM between individual animals, each temporal series was sampled at 32 points and expressed as a percentage of the cardiac cycle. Flow curves representing carotid and sinus flows were calculated using the mean flow values from the left and right sides of the brain

The mean heart rate was 340 BPM, with a standard deviation of 70 BPM. Figure 4 shows the flow curves of each vessel of interest obtained from our group of rats, with the exception of one rat whose lateral sinuses velocity images presented a low signal-to-noise ratio. Table 1 summarizes the mean and maximum values of the measured blood velocity and flow. These measurements revealed that the ICA had a higher peak velocity value than the ECA, BA, and sinuses. There was no significant difference between the mean measures of cerebral inflow (combined ICA and BA flows) and cerebral outflow (combined left and right transverse sinuses flows). The mean ICA flow (9.6 ± 3.4 ml/min) was higher than mean ECA flow (5.7 ± 2.4 ml/min), *p* < 0.003. However, the PI of the ICA (2.02 ± 0.24) was lower than that of the ECA (3.73 ± 1.19), *p* < 0.004. Furthermore, the PI of the ICA (2.02 ± 0.24) was higher than that of the transverse sinuses (0.37 ± 0.19), *p* < 0.002.

**Table 1:** Mean (±SD) values of velocity and flow measured in our rat group

	Velocity (cm/s)		Flow (ml/mn)	
	Mean ± SD	Maximum ± SD	Mean ± SD	Maximum ± SD
Basilar artery	9.6 ± 3.7	17.1 ± 7.0	2.0 ± 0.6	3.6 ± 1.2
Left ICA	9.1 ± 4.2	20.8 ± 8.5	4.9 ± 2.0	11.3 ± 4.8
Right ICA	10.2 ± 3.9	24.6 ± 9.4	5.0 ± 1.3	11.9 ± 2.9
Left ECA	5.5 ± 3.1	17.5 ± 6.3	2.9 ± 1.4	9.3 ± 2.3
Right ECA	5.4 ± 2.2	23.5 ± 12.5	2.9 ± 1.3	11.4 ± 5.2
Left transvers sinus	4.0 ± 2.1	4.8 ± 2.3	5.1 ± 3.2	6.1 ± 3.3
Right transvers sinus	4.4 ± 1.8	5.1 ± 1.9	5.4 ± 1.7	6.3 ± 1.9

*ICA* internal carotid artery, *ECA* external carotid artery

**Fig. 5:**
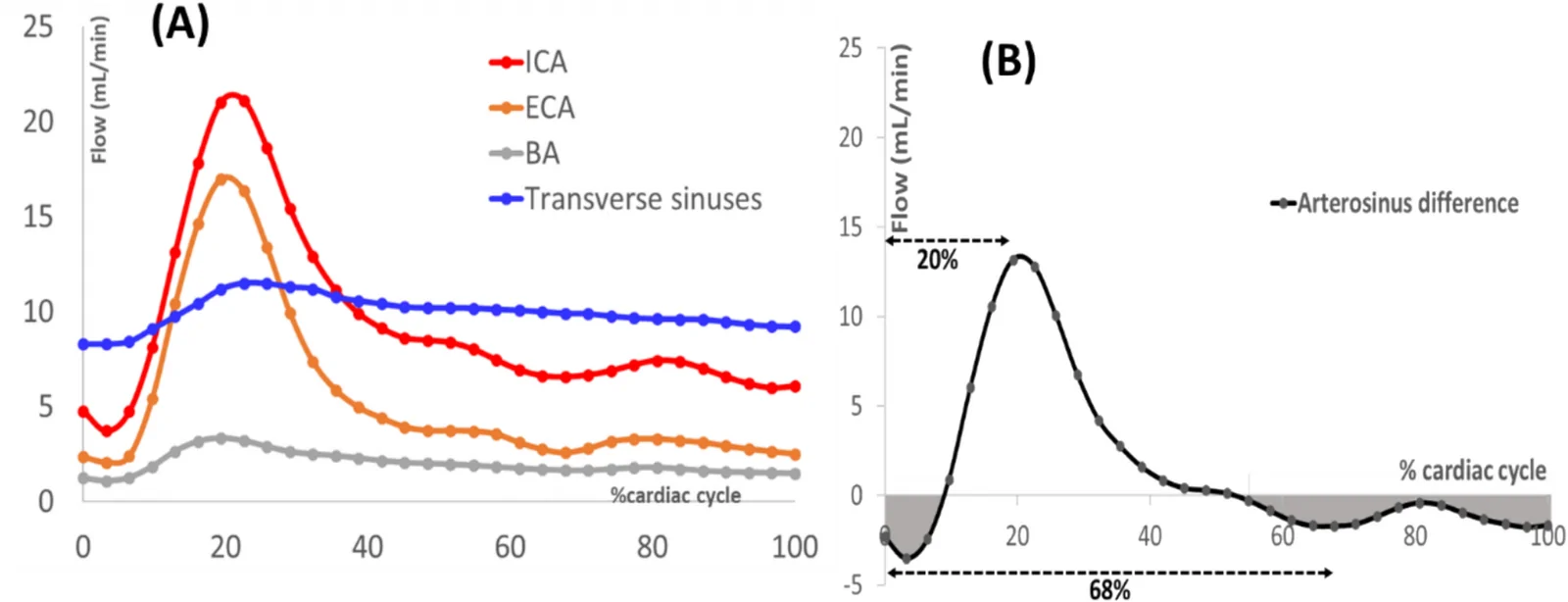
**A** Group-average flows for the internal carotid artery (ICA), external carotid artery (ECA), basilar artery (BA), and transverse sinuses are shown. **B** The curve representing the arterio-sinus flow difference is represented. The gray area located between this curve and the x-axis represents times when the flow of the transverse sinuses dominates over that of the ICA flow. The times at which the flow peak reflecting the 20 and 68% systolic and diastolic cardiac phases can be seen, respectively

No significant difference was found between mean flow measured from the left and right sides of the brain, for both arterial and sinus flows, in our group of rats. Figure 5A shows plots of the average group flow curves for the vessels of interest. Although there was no significant difference in the mean flows of the ICA/ECA and the transverse sinuses, the rapid velocity changes related to the systolic and diastolic cardiac phases were more visible in the arterial system than in the venous system. Figure 5B shows the flow curve representing the arterio-sinus difference. The gray area between this curve and the x-axis represents times when flow of the transverse sinuses is dominant than that of the ICA flow. Our results also demonstrate that the pulsatility of the rat arterial system is markedly higher than that of the sinus system.

## Discussion

In this study, our primary focus was on validating velocity measurements at 7T using a Y-shaped flow phantom. Two pump regimes were used, with flow rates of 40 and 50 ml/min, to produce low, medium, and high flow velocities in the three phantom tubes. These velocities corresponded to ranges of physiological blood flow in the rat brain. Furthermore, our experiment involved optimizing the flow sequence using phantom tubes with a relatively small inner diameter. It is known that PC-MRI velocity assessment at low flow rates is less sensitive for small-caliber vessels, as data acquisition with acceptable spatial resolution and signal-to-noise ratio is required [21]. Using tubing that had a cross-sectional area comparable to that of the caliber of the major rat cerebral arterial and venous vessels, we demonstrated PC-MRI capability to quantify flow velocities with an excellent repeatability (reflected by a small %CV) and a relatively good accuracy of the velocity measurements. At a reference flow of 50 ml/min, the repeatability and accuracy coefficients for the flow measurements were slightly higher than those at 40 ml/min. However, the flow distribution through the three branches of the bifurcation was not proportional to the tube lumen areas, particularly in the tube with the smallest inner diameter, where the flow was considerably lower than that in the other two tubes. This may reflect a marked increase in flow resistance when the inner radius of the tube decreases, as a consequence of the fourth power relationship between flow and diameter in Poiseuille’s law. To our knowledge, studies validating PC-MRI flow measurements at high-field animal MR scanner were usually performed under non-pulsatile flow conditions [22].

Relevant blood flow information can be obtained from the cine PC-MRI, a technique that has been widely applied to various human cerebrovascular pathologies [23–25] as well as to neurodegenerative diseases [26, 27]. Our results provide velocity measurements and quantitative blood flow values in the major feeding and draining cerebral vessels of rats, which may be useful for obtaining reference values for rat cerebral PC-MRI velocimetry. Our PC-MRI velocity measurements for ICA and BA are within the ranges of values measured by transcranial Doppler sonography technique in rats [7]. However, caution is required when comparing measurements between the two techniques due to the different experimental conditions. We found that the pulsatility of the ECA was higher than that of the ICA, despite the fact that its mean blood flow was lower. These differences in flow and pulsatility may reflect differences in ICA and ECA circulations. In rats, the ECA branches into the occipital artery, the superior thyroid artery, the lingual artery, and the external maxillary artery [28] which supply blood to territories that are mostly extracerebral regions such as mastication and neck muscles, the pharynx, the tongue, the salivary glands, and the face. In contrast, the vascular territories of the ICA include external parts of the brain (through the pterygopalatine artery), but predominantly supply cortical and deep brain structures (such as the basal ganglia and thalamus), the major blood sources of which are part of the circle of Willis [29]. The cerebral arterioles and capillary bed play an important role in maintaining local blood flow and adequate perfusion pressure within the normal physiological ranges. A decrease in arterial caliber through these cerebral networks may explain why ICA pulsatility was attenuated compared to that of ECA pulsatility without blood flow being altered. This is because the high-density small vessels in the ICA territory can compensate for an increase in vascular resistance. Therefore, a decrease in ICA pulsatility may contribute to the stabilization of intracranial pressure. Other findings in literature are consistent with this, indicating that cerebral perfusion pressure is more stable in the ICA territory than in the ECA bed in rats [30].

While previous PC-MRI studies in rats have been focused on measuring blood flow in the arterial system [11, 12], it is also important to quantify flow and its properties in the venous-sinus system. Our study provided quantitative velocity and flow measurements in the transverse sinuses, which drain the cortical blood flow through the superior sagittal sinus. The results revealed lower flow pulsatility in the transverse sinuses than in the arterial network, indicating differences in flow dynamics between these two networks. One possible explanation for this result is that the compliance of the arteriole and capillary beds in rats likely dampens a large part of the arterial pulsations. Figure 5B shows a negative transverse sinus flow relative to the ICA flow during the early systolic and end diastolic cardiac phases. This reflects relatively constant transverse sinus flow (low pulsatility) during systole and diastole, which enables cerebral inflow and outflow to remain at the same level despite the arterial fluctuations.

The findings of this study could have numerous implications, particularly with regard to investigating cerebrospinal fluid (CSF) dynamics in cases of hydrocephalus and the glymphatic system. PC-MRI studies conducted in rat models of hydrocephalus have measured CSF flow without considering the dynamics of vascular fluids [31]. An imbalance in the dynamic interaction between changes in cerebral blood flow and CSF oscillations in the Sylvius aqueduct was reported in elderly population [26]. This provides evidence of interaction between cerebral blood flow and CSF dynamics. This is consistent with the findings of Bateman [3] in human healthy subjects, which demonstrate that all arterial pulsations can be accounted for by venous flow and the volume of CSF exiting the intracranial system. More recently, emerging evidence suggests that cranial CSF dynamics facilitate an efficient clearance of interstitial solutes and waste from the brain parenchyma [32], by exchanging them through the perivascular routes of deep arterioles [33]. This microcirculation of the CSF flow is an important component of glymphatic system function and is facilitated by the transmission of cerebral arterial pulsations to the microvasculature [34].

Our flow data were acquired with a reasonable spatial and temporal resolutions relative to the diameters of the vessels of interest. These measurements may have preclinical relevance because, in addition to helping to better understand rat models of cerebral vascular diseases, they can be useful for choosing the optimal VENC sequence parameter for cerebral PC-MRI velocimetry.

This study has some limitations. First, it was conducted on female Sprague–Dawley rats, which somewhat limits the generalization of these findings. Second, this study did not include a sex-matched subgroup to limit variability induced by the rapid brain development of male rats [35]. Furthermore, the influence of age on our flow measurements could not be analyzed due to the small size of the group.

Other practical considerations relevant to flow measurements under anesthesia should also be considered. Most of anesthesia substances exert physiological effects on the cardiovascular system and cerebral blood flow to some extent. The vasodilator properties of isoflurane are well known [36], but low doses can minimize its cardiodepressive effects and produce stable mean arterial pressure and heart rates [37]. Furthermore, dose-dependent increases in cerebral blood flow have been also observed with isoflurane in rats [38]. Despite these limitations, the majority of current MRI studies investigating cerebral flow in rodents have been conducted using isoflurane, because it enables rapid induction, precise control of concentration, and rapid recovery.

Regarding the phantom study, an ideal flow phantom should include compliance, such as that used to model the aortic arch [39]. Defined as the change in volume per unit change in pressure, compliance may influence vascular hemodynamics and it is also considered as a marker of vessel stiffness. In our experiment, we detected the pump cycle through flow pulsatility using a thin-walled latex balloon that was integrated into the flow loop upstream from the phantom. The phantom’s tubing walls were relatively rigid compared to the balloon. Therefore, changes in pressure-volume due to flow pulsatility affected the balloon more than the tubing of the phantom. The inflow waveform was dampened by the balloon’s compliance before entering the phantom.

In conclusion, the application of these measurements on preclinical cerebrovascular diseases are promising. These results could provide further insights on cerebral blood flow dynamics and physiology of rat disease models in preclinical studies.

## Data Availability

The data that support the findings of this study are available from the corresponding author upon reasonable request.
